# Compound Lever Forceps

**Published:** 1851-07

**Authors:** 


*Compound Lever Forceps.*—We are in receipt of an instrument from Dr. J. C. Burch, of Evansville, Indiana, which he calls a *Compound Lever and Elevating Forceps;* it has some good qualities, and we propose giving a close description of it, when the proper cuts can be procured; until which time, we must beg the indulgence of Dr. B., as well as our readers.

